# Professional identity development among male psychiatric nurses in China: a qualitative study

**DOI:** 10.1186/s12912-026-05364-7

**Published:** 2026-09-21

**Authors:** Danni Chi, Lijun Zhao, Aiqiang Chi, Nan Chen, Yingjing Bao, Wenbo Chen, Jianing Dai, Baoqin Yang

**Affiliations:** Department of Psychiatry, Zhejiang Key Laboratory of Drug Addiction & Brain Health, Affiliated Kangning Hospital of Ningbo University (Ningbo Kangning Hospital), Ningbo, China

**Keywords:** Male nurses, Psychiatric nursing, Professional identity, Gender stereotypes, Qualitative research

## Abstract

**Background:**

Men remain underrepresented in nursing and often face gender stereotypes, occupational stigma, and challenges related to professional identity. These issues may be particularly pronounced in psychiatric nursing, where care involves therapeutic relationships, emotional labor, long-term patient contact, and exposure to aggression. This study explored how professional identity developed over the careers of male psychiatric nurses in China.

**Methods:**

A qualitative study was conducted with 16 male psychiatric nurses from a specialist psychiatric hospital in China. Data were collected between October and December 2025 through semi-structured interviews and analyzed using reflexive thematic analysis.

**Results:**

Three themes were developed. *Stumbling into commitment* described how participants entered psychiatric nursing through pragmatic or deliberate routes, encountered early shock and a double stigma relating both to men in nursing and to psychiatric work, and gradually settled into the work. *Growing through relationships* showed how professional growth developed through patients, colleagues, and teams, as participants moved from task completion toward relational and preventive judgment. *Strength in caring* captured how physical strength, often expected of male nurses, could be useful in acute risk situations when integrated with restraint, protection, clinical judgment, and relational care. Participants described strong commitment and pragmatic stability, alongside a contrasting account of occupational fatigue.

**Conclusions:**

Male psychiatric nurses’ professional identity developed gradually and unevenly through career experiences involving pragmatic entry, early shock and strain, relational learning, and strength in caring. Nursing managers should support early adaptation, occupational safety, mentorship, relational competence, and long-term career sustainability.

**Supplementary Information:**

The online version contains supplementary material available at https://doi.org/10.1186/s12912-026-05364-7.

## Introduction

Professional identity in nursing has been conceptualized as nurses’ understanding of themselves as members of the profession, including the values, knowledge, roles, and standards that guide their practice [1]. Professional identity develops gradually through education, clinical experience, workplace relationships, and reflection on practice, as nurses interpret these experiences and refine their sense of professional role and value [2]. The professional environment, therefore, provides an important context for identity development. For male psychiatric nurses, this environment may be especially complex. They work as a gender minority in a profession traditionally associated with women and in a specialty affected by stigma toward mental illness and psychiatric care [3, 4]. Stigma refers to a social process in which labeling and stereotyping contribute to separation, status loss, and discrimination within unequal relations of power [5]. These conditions may shape how male psychiatric nurses are perceived by patients, colleagues, healthcare managers, and the wider public, influencing both their workplace experiences and how they understand their place within the profession.

Gender stereotypes are widely shared beliefs about the attributes, behaviors, and social roles considered appropriate for women and men, shaping how individuals are perceived and treated in occupational settings [6]. In nursing, these stereotypes are closely related to the traditional representation of nursing as women’s work. Men may experience doubts about their suitability for caring roles, difficulties gaining acceptance, and social isolation. Their visibility and presumed physical strength may also bring particular responsibilities or career opportunities, illustrating the complex influence of gender on their professional experiences [7]. More specifically, male nurses may be questioned about their caring ability, career choice, and sexuality. They may also be excluded from intimate care while being expected to undertake physically demanding tasks, manage aggression, or maintain safety [4]. These stereotypes can shape both how men are accepted within nursing and the professional roles assigned to them. In the Chinese context, nursing has traditionally been associated with women and with attentiveness and caring, whereas broader social expectations have positioned men as physically strong and responsible for family income [8]. These expectations reflect masculinity stereotypes within the broader pattern of gender stereotyping and provide a sociocultural context for understanding male nurses’ professional experiences [7, 8]. Existing reviews have synthesized men’s experiences in nursing across diverse specialties and cultural contexts [4, 7], but limited attention has been given to how these experiences shape professional identity development in psychiatric nursing.

Psychiatric nursing occupies a distinctive position within this pattern. Although men remain a small minority of the nursing workforce in China, they appear to be relatively better represented in psychiatric nursing: a national survey of 9,256 psychiatric nurses from 41 psychiatric hospitals across 29 provinces found that 20.7% were men [9]. This indicates that men are more strongly represented in psychiatric nursing than in nursing overall. One possible explanation is that recruitment and work allocation are influenced by gender stereotypes that portray men as suited to managing acute risk, physical intervention, and demanding shift work [7, 8]. Such stereotypes may facilitate men’s entry into psychiatric nursing while also directing them toward a limited range of roles defined by physical capacity.

In psychiatric nursing, negative beliefs about mental illness and psychiatric services may extend to the nurses working in these settings. Psychiatric nurses have been perceived as less skilled, logical, dynamic, and respected than nurses in other specialties, suggesting that psychiatric nursing may hold a devalued position within the profession [3]. Such stigma may also influence how nurses understand their professional identity, especially when their specialist role is poorly defined or receives limited institutional recognition [10]. The low visibility and perceived status of mental health nursing have further been associated with difficulties in recruitment and retention [11]. Stigma, low visibility, and limited institutional recognition may therefore affect psychiatric nurses’ sense of professional legitimacy and their future within the specialty.

Research specifically concerning male psychiatric nurses remains limited. An ethnographic study in psychiatric settings showed that male nurses negotiated their professional identity through everyday efforts to reconcile caring practice with prevailing views of masculinity, indicating that gender stereotypes were embedded in routine clinical work rather than confined to external social attitudes [12]. However, the study was small and reflected a particular historical and cultural context. A recent scoping review of 24 publications confirmed that the wider evidence base remains fragmented. Existing studies have focused mainly on characteristics attributed to male psychiatric nurses and on factors affecting recruitment and retention, including meaningful patient care, team support, workplace violence, stigma, and limited career development opportunities [13]. Many of these studies were relatively old, included mixed-gender samples, or examined isolated aspects of work experience. Consequently, little is known about how male psychiatric nurses come to understand their professional role and value as their careers unfold.

The development of professional identity among male psychiatric nurses may have particular features in China. Although occupational status, income, and family responsibilities influence men’s career choices in many countries, studies in China have also found that some men entered nursing through university admission and major allocation processes after they did not meet the score requirements for their preferred programs [8, 14]. Combined with the persistent association of nursing with women, the relatively low perceived occupational status of nursing, and traditional beliefs about men’s social position [8, 15], this may make men’s presence in nursing more socially conspicuous and more open to questioning. Interviews with 25 male nurses and two hospital administrators showed that public prejudice and the low social status of nursing shaped men’s entry into and continuation in the profession, although greater visibility sometimes brought career opportunities [15]. A study of male nursing students and junior male nurses further found that professional identity weakened across education and was lowest during the early years of practice, with gender stereotypes and heavy workloads contributing to this pattern [16]. However, existing research from China has focused on general nursing settings and early career stages, leaving professional identity development among Chinese male psychiatric nurses across their careers insufficiently understood. This study aimed to explore how male psychiatric nurses’ professional identity developed over the course of their careers.

## Methods

### Study design

This study adopted a qualitative design and used semi-structured interviews to explore how male psychiatric nurses’ professional identity developed through their career experiences [17]. A qualitative approach was appropriate for examining complex experiences shaped by clinical, organizational, and social contexts that cannot be adequately captured by predefined quantitative measures. Semi-structured interviews were used to elicit in-depth accounts while allowing flexibility to pursue issues raised during the interviews [17]. Data were analyzed using Braun and Clarke’s reflexive thematic analysis (RTA) [18]. The analysis was guided by a data-driven and inductive orientation, with codes and themes developed through close engagement with the dataset rather than a pre-existing coding framework [19]. Throughout the analytic process, emphasis was placed on analytic reflexivity and the active, interpretive role of the researcher in theme development.

### Setting and context

This was a single-center study conducted at a tertiary-grade A specialist psychiatric hospital in China. Tertiary Grade A represents the highest level in the Chinese hospital classification system. The hospital is a large specialist mental health institution providing psychiatric inpatient and outpatient services. In China, psychiatric nurses qualify through general nursing education and nurse registration. Pre-registration nursing programs may include coursework in psychiatric nursing and, depending on the institution, a short psychiatric clinical placement. More specialized knowledge and clinical competence are subsequently developed through workplace training and experience after entering psychiatric practice.

### Participants and sampling

Convenience sampling was used to recruit male psychiatric nurses who were readily accessible through the study hospital and its professional psychiatric nursing communication group [17]. Eligible participants were male registered nurses who worked in psychiatric nursing at the study hospital, had at least 1 year of psychiatric nursing experience, and could communicate in Mandarin. Recruitment information was circulated through the professional communication group. Nurses who were interested in participating registered by scanning the QR code on the recruitment poster, completed a brief demographic questionnaire, and provided informed consent through a secure mobile platform. Nurses who met the eligibility criteria were then contacted individually by the interviewers to arrange an interview. Participation was voluntary, and participants received no financial compensation or expense allowance. All participants held either a college diploma in nursing or a bachelor’s degree in nursing.

An initial sample range of 15 to 20 participants was planned. The first round of recruitment resulted in 16 eligible nurses who consented to participate and completed an interview. The research team then reviewed the sample composition and discussed whether a second recruitment round was required. Participants ranged in age from 23 to 50 years (M = 32.81) and had between 1 and 27 years of psychiatric nursing experience (M = 10.56). The research team also reviewed the breadth and depth of the completed interview accounts and judged that the dataset provided sufficient variation and detail to address the study aim, and a second recruitment round was unnecessary.

### Ethical considerations

Ethical approval for the study was obtained from the institutional ethics committee prior to data collection (Approval No. NBKNYY-2024-LC-64). All participants provided informed consent before participation and were informed that participation was voluntary and that they could decline to answer any question, discontinue the interview at any time, or withdraw from the study within two weeks after the interview.

All interviews were transcribed verbatim in Mandarin and de-identified within two weeks of completion. During transcription, all identifying information was removed. Participants were randomly assigned numerical identifiers (N01–N16) for reporting purposes. The identifiers did not correspond to interview order, demographic characteristics, or institutional roles. To further reduce the risk of deductive disclosure in this small, single-center sample, participants’ age and years of psychiatric nursing experience were reported in grouped categories when presented alongside individual extracts. After de-identification, individual interview data could no longer be linked to specific participants and therefore could no longer be withdrawn.

Confidentiality was maintained throughout the research process. Signed consent forms and identifying records were stored separately from the interview recordings, demographic data, and de-identified transcripts. Electronic files were password-protected and accessible only to authorized members of the research team. Audio recordings were deleted after the transcripts had been checked for accuracy.

### Data collection

Data were collected through semi-structured interviews conducted between October and December 2025. The semi-structured interview guide was developed specifically for this study based on the study aims and relevant literature (Supplementary File 1). The draft guide was refined through discussion within the research team, drawing on its nursing, nursing management, clinical psychology, and qualitative research expertise. It focused on participants’ pathways into psychiatric nursing, changing views of the profession over time, challenges and supports encountered during different career stages, and future career plans. Follow-up questions were used to clarify and deepen participants’ responses where appropriate. No pilot interviews were conducted.

Interviews were conducted by two trained frontline psychiatric nurses, one male (AC) and one female (NC). Both interviewers had received training in qualitative interviewing before the study. Interviews took place outside working hours in locations chosen by participants to maximize privacy and comfort. Depending on participant preference and feasibility, interviews were conducted either face-to-face or by telephone. Face-to-face interviews were conducted in private locations selected in consultation with participants. Telephone interviews were used when preferred by participants or when an in-person meeting was less feasible. The same interview guide and flexible probing approach were used in both formats. No clear differences in relevance or depth were identified during analysis.

All interviews were conducted in Mandarin, lasted 28–44 min (M = 34.87 min), and were audio-recorded with participants’ permission. The original Mandarin transcripts were used throughout coding and theme development. Participants’ identities and personal information were treated as confidential throughout the study. Complete anonymity could not be guaranteed because the interviewers saw participants during face-to-face interviews or heard their voices during telephone interviews. Transcripts were de-identified and participants were referred to by codes (N01–N16), as described in the *Ethical considerations* section. Quotations selected for the manuscript were initially translated into English using ChatGPT. The first author, who completed postgraduate education in an English-speaking country and has extensive experience in English academic writing, compared each translation with the original Mandarin extract and revised the wording where necessary. Particular attention was given to preserving participants’ intended meanings, tone, and contextual nuances.

### Data analysis

Formal coding and theme development began after all 16 interviews had been completed. The transcripts were analyzed inductively using Braun and Clarke’s six-phase process for reflexive thematic analysis [19, 20]. In the first phase, members of the analytic team (DC, AC, NC, and LZ) read the transcripts repeatedly to become familiar with individual accounts and the dataset as a whole and recorded initial analytic observations. In the second phase, initial codes were generated across the dataset with close attention to participants’ meanings and experiences. The first author coordinated the coding process and used NVivo 15 to organize codes together with their associated data extracts, allowing each coded extract to remain linked to its original transcript and surrounding context.

In the third phase, codes and their associated extracts were examined to generate initial themes, each organized around a shared idea or central organizing concept. Where an initial theme contained distinct but connected aspects of its central organizing concept, provisional subthemes were developed to structure these aspects. In the fourth phase, the initial themes and subthemes were reviewed against both the coded extracts and the complete dataset to assess their coherence, boundaries, distinctions, relevance to the study aim, and fit with participants’ accounts. During this process, themes and subthemes were combined, separated, reorganized, or renamed where necessary, and less typical or divergent accounts were considered when refining their scope.

In the fifth phase, the themes and subthemes were further refined, defined, and named, with attention to the central organizing concept and analytic focus of each theme. In the sixth phase, the analytic narrative was developed and representative extracts were selected from the coded material to illustrate and support the interpretations presented in the Results. Each selected extract was checked against its original transcript to ensure that it was presented in context. Reflexive notes and analytic memos were used throughout to document developing interpretations, examine analytic assumptions, and support the refinement of themes. Table 1 presents illustrative examples of the progression from preliminary codes to subthemes and themes. Although described in six phases, the analysis was recursive, with movement between coding, theme development, review, and analytic writing.

**Table 1:** Development of themes from representative preliminary codes

Theme	Subtheme	Examples of preliminary codes
Stumbling into commitment	Pragmatic entry	Seeking secure employment, family influence, no clear career plan, deliberate choice of psychiatric nursing
	Early shock and strain	Feeling overwhelmed, shock at patient aggression, fear after violence, feeling misunderstood after violence
	Negotiating double stigma	Gender stereotypes about nursing, stigmas surrounding psychiatric work, concealing one’s job, openly identifying as a psychiatric nurse
	Commitment through accumulation	Growing familiarity, gaining confidence, patient trust, settling into the work, fatigue, reduced motivation
Growing through relationships	Developing relational understanding	Looking beyond symptoms, reading patient cues, aggression as illness-related, reflecting on prevention
	Learning with the team	Observing senior nurses, seeking guidance, support after incidents, team coordination
	Finding meaning in care	Small acts of care, patient gratitude, helping families, learning from difficult encounters
Strength in caring	Physical strength as a resource	Physical advantage, calmness under pressure, relied on in emergencies; expected to step forward despite fear
	Protective strength	Avoiding retaliation, de-escalation before force, protecting colleagues and patients, staying prepared for unpredictability
	Strength with relational care	Communication before physical intervention; firm but gentle approach; relational disadvantage with female patients; self-control during aggression

### Research team and reflexivity

The first author (DC) is a clinical psychologist with a medical background and doctoral training in psychology, with experience in qualitative and mixed-methods research. She led the data analysis and interpretation. AC, a male frontline psychiatric nurse, and NC, a female frontline psychiatric nurse, conducted the interviews and participated in data interpretation and theme development. LZ, a senior nurse with more than 20 years of nursing experience, contributed to the interpretation and refinement of the themes from clinical and managerial perspectives.

The interviewers were both frontline psychiatric nurses and did not hold managerial roles. They worked in different departments from the participants, had no direct supervisory relationships with them, and had no frequent routine contact or known conflicts of interest. This helped to reduce potential power imbalance and supported participants in speaking more freely about their experiences. The research team recognized that interpretation was actively shaped by their disciplinary backgrounds, clinical experiences, and gender positions. Throughout the analytic process, these complementary perspectives were treated as interpretive resources and were continually reflected upon during theme development. Different interpretations were discussed openly, with attention to how psychological, nursing, gender, and managerial perspectives informed the developing analysis. Rather than seeking a single “correct” interpretation, the team used these discussions to deepen understanding of participants’ experiences and to refine theme boundaries, central organizing concepts, and the final thematic structure.

### Research quality and reporting

The study was reported in accordance with the Consolidated Criteria for Reporting Qualitative Research [21]. Methodological coherence was supported by aligning the study aim, qualitative design, semi-structured interviews, inductive orientation, and RTA. Credibility, dependability, confirmability, and transferability were addressed through the reflexive, transparent, and contextually grounded procedures described above, together with verbatim quotations linking the interpretations to participants’ accounts [22].

## Results

**Fig. 1:**
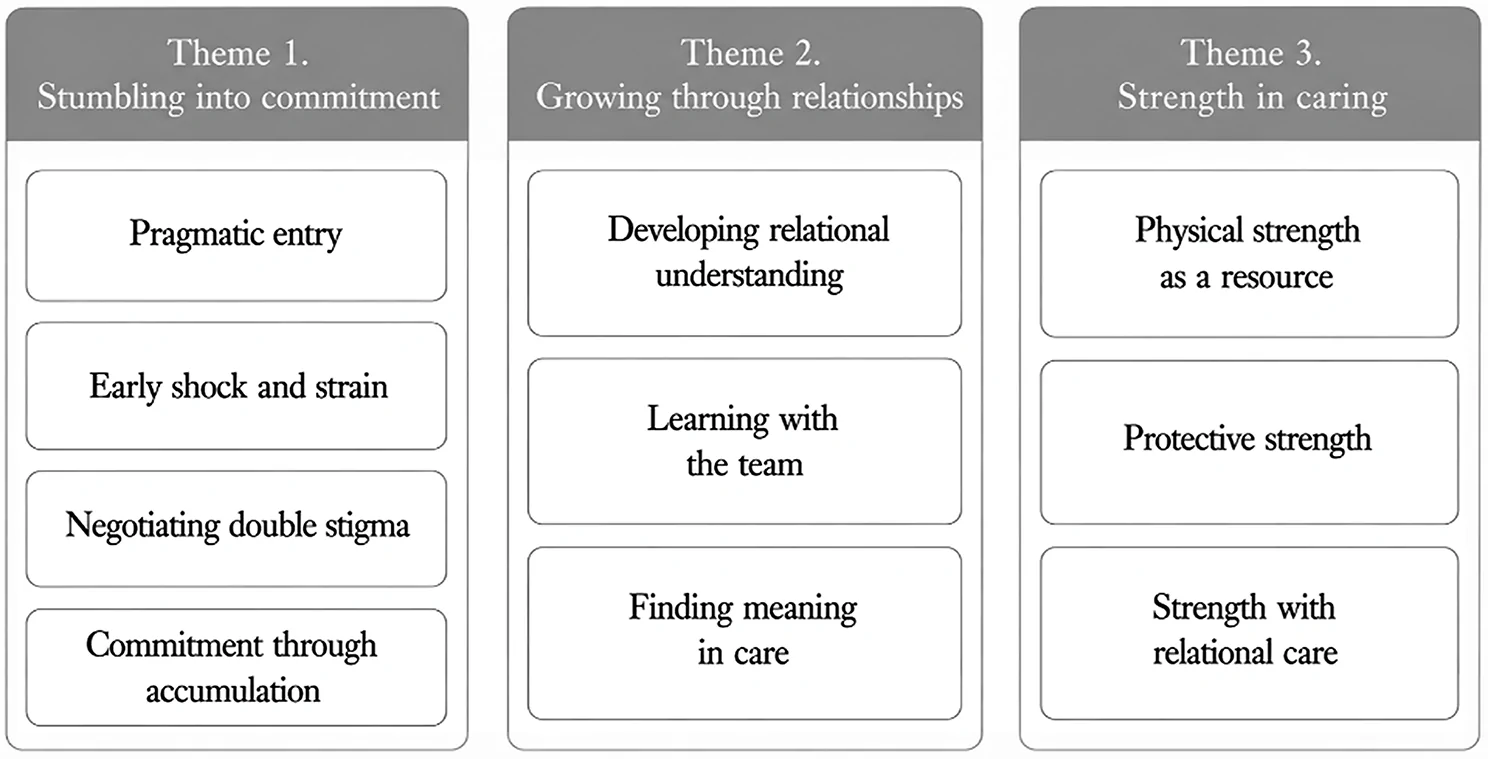
Thematic map of the themes

Three themes were developed from the data (Table 1; Fig. 1): *stumbling into commitment*, *growing through relationships*, and *strength in caring*. These themes describe participants’ pathways into psychiatric nursing and their varying degrees of professional identification and commitment, their professional growth through relationships with patients and colleagues, and their understanding of physical strength as a situated resource in care while responding to expectations placed on male nurses. Theme 1 traces a broadly chronological movement from pragmatic entry toward different degrees of commitment and professional identification, while Themes 2 and 3 examine two interrelated processes, relational growth and strength in caring, that unfolded across this movement without constituting sequential stages. The three themes show how these processes jointly shaped how participants came to understand themselves as psychiatric nurses.

### Theme 1. Stumbling into commitment

This theme describes how participants’ professional self-understanding developed following pragmatic or uncertain entry into psychiatric nursing. Early shock and the double stigma of being men in nursing and working in psychiatry challenged the legitimacy and value they attributed to the role. Through accumulated experience, some developed stronger professional identification and commitment, whereas others remained pragmatic or became fatigued.

#### Pragmatic entry

Participants entered psychiatric nursing through practical rather than idealized pathways. Employment opportunity, exam results, family advice, and job stability were often more immediate than a strong motivation. Participant N01 described his entry straightforwardly:

I had just graduated and was looking for a job. I happened to see that the hospital was recruiting, so I applied. At that time, I did not think too much. I just thought, get in first, pass the exam first, and then see. (N01, 31–40 years, over 15 years’ experience)

His account captures a broader pattern in which participants initially approached psychiatric nursing as an available and secure job rather than as a career with which they already strongly identified.

Similar pragmatic considerations appeared in other accounts. Some participants linked their entry to financial necessity, classmates’ choices, or family advice. These routes suggest realistic navigation of the labor market rather than indifference or passivity. For many, commitment did not precede entry into psychiatric nursing but developed gradually through working in the ward.

More deliberate routes were also described. Participant N10, for example, recalled: *Nursing was the career I had always wanted. I had also been interested in psychiatry for a long time* (N10, 21–30 years, 1–5 years’ experience). Even among those with stronger initial interest, their understanding of their professional role continued to be shaped by clinical practice.

#### Early shock and strain

Early practice was frequently described as demanding in ways that exceeded participants’ prior expectations. Many participants first encountered psychiatric nursing not as a stable professional role but as a site of unpredictable risk and emotional strain. For Participant N11, the first experience of protective restraint made the difference between psychiatric and general nursing sharply concrete:

The first major shock was probably the first time I carried out a protective restraint on a patient. The patient suddenly had an acute episode. During my student placement, I had never personally carried out a protective restraint, so the atmosphere felt very tense the first time. I was not on duty alone. We called security to help hold him so that he would not injure himself by struggling too forcefully, and we then applied the restraints. He was very agitated, shouting, struggling forcefully with his arms, swearing, and spitting. It felt dangerous. At that moment, I immediately felt the biggest difference between psychiatric nurses and nurses in general hospitals. (N11, 21–30 years, 1–5 years’ experience)

This early shock could also become a sustained strain. Participant N16 described the pressure of transferring from a rehabilitation ward to an acute male unit, where violent incidents were more frequent and less predictable: *The pressure felt very heavy. At that time*,* I was so tense and anxious that my hair started falling out* (N16, 31–40 years, 6–10 years’ experience). In N16’s account, the strain of this transition was experienced as intense emotional distress accompanied by hair loss.

For Participant N12, the physical injury was compounded by a sense of injustice. After being struck by a patient without warning and sustaining a facial injury, he recalled: *I felt very wronged. I was treating him and trying to help him recover* (N12, 31–40 years, 11–15 years’ experience). His response points to a difficult tension in psychiatric nursing: caring for patients while remaining vulnerable to harm from them. These accounts suggest that early adaptation required more than mastering ward routines; participants had to learn to tolerate uncertainty, risk, and emotional demand.

#### Negotiating double stigma

Participants encountered two related forms of stigma: gender stereotypes about men in nursing and occupational stigma surrounding psychiatric work. These experiences shaped how some participants presented their work to others. Participant N05 recalled:

At first, I was reluctant to tell others that I was a male nurse. People thought that nurses in hospitals were all women, and they might be prejudiced against or distrustful of men. Later, when I began working at a psychiatric hospital, I did not want to tell people that I was a psychiatric nurse. When people asked where I worked, I would say an ordinary hospital. I was afraid people would think that, because I stayed with psychiatric patients, I might also be abnormal. (N05, 21–30 years, 11–15 years’ experience)

Participant N05’s account shows how gender stereotypes and psychiatric stigma influenced whether he disclosed his occupational role to others.

Over time, this discomfort was often reduced. Some participants became more accepting of their work as they gained experience, while others felt that male nurses had become more visible and accepted within psychiatric hospitals. Even so, stigma did not disappear entirely. In interactions outside the hospital, participants still had to negotiate assumptions about gender, nursing, and mental illness. These experiences affected the legitimacy and social value participants attributed to their work, showing how professional self-understanding was shaped by both personal experience and others’ responses.

#### Commitment through accumulation

Participants did not describe a single moment that transformed them into committed psychiatric nurses. Staying was more often cumulative: work became familiar, risks more manageable, and competence gradually increased. Participant N06 reflected: *After doing this work for so many years*,* I gradually became used to it and became more competent. I no longer spend much energy thinking about other careers* (N06, 31–40 years, 11–15 years’ experience). For him, commitment was less a felt passion than a gradual settling into the work through familiarity and accumulated capability.

Employment conditions also shaped this process. For some participants, job stability, permanent posts, and financial security made leaving less likely. Professional identification developed alongside practical constraints, not despite them. Staying was, in this sense, as much a matter of structure as of personal adaptation.

Relational experiences could deepen commitment in a different register. Participant N13 recalled a patient’s direct expression of attachment: *A child told me*,* ‘I know you are leaving*,* but I really like you. I hope you can stay here.’ That was why I finally chose to stay in that ward* (N13, 21–30 years, 6–10 years’ experience). Such moments gave psychiatric nursing emotional significance and helped participants experience their presence as meaningful. Commitment, across these accounts, accumulated through competence, stable employment, and relational recognition, not through any single vocational turning point.

This cumulative pattern did not characterize every account. N14 described pronounced occupational fatigue and reduced motivation for further professional development. Reflecting on his future career, he said, “*I feel very fatigued now. I used to want to obtain an associate senior title*,* but now I no longer want to pursue it.*” (N14, 41–50 years, over 15 years’ experience). In this account, continued employment was marked by disengagement rather than an accumulating commitment to psychiatric nursing.

### Theme 2. Growing through relationships

Theme 2 describes how relationships with patients, families, and colleagues reshaped participants’ understanding of what psychiatric nursing involved and what made their work professionally valuable. The three subthemes show how they developed a relational understanding of patients that informed clinical judgment, learned through team interaction, and found meaning in care. These experiences expanded participants’ professional self-understanding beyond task completion toward a view of psychiatric nursing as interpretive, relational, and morally demanding. This change was gradual and uneven, shaped by accumulated experience and repeated encounters with patients’ needs, risks, and responses. Encounters involving aggression were especially salient because they prompted some participants to reconsider how they understood patients and how they responded in care.

### Developing relational understanding

Professional growth involved developing a relational understanding of patients’ behavior, distress, and needs. This understanding also informed how participants anticipated risk and adjusted their responses in practice. Some participants first learned to reframe aggression as illness-related rather than personal. Participant N07 recalled being told that *everyone gets hit* and that *patients were ill* and *not doing it deliberately* (N07, 21–30 years, 1–5 years’ experience). The reframing helped him continue care after painful encounters, though without reflection and prevention, it risked normalizing violence rather than addressing it.

Clinical maturity, however, required more than tolerance. Participant N09, reflecting on a staff injury, emphasized preventive judgment:

After that incident, we reflected on what we should pay attention to at work, what loopholes there were, and what we had not done well enough. It is like building a house. You have to lay the foundation firmly (N09, 21–30 years, 1–5 years’ experience).

His account points to a form of growth oriented toward anticipating risk and strengthening team routines, not absorbing harm through individual toughness.

Growth also involved moving from task completion to relational care. Participant N15, who had entered psychiatric nursing from an emergency setting, recalled:

After spending more time with long-stay patients, I felt that this was not enough. For psychiatric patients, what matters more is care for the mind. They are a vulnerable group; many things they cannot tell others, and others cannot understand (N15, 31–40 years, 6–10 years’ experience).

His account marked a change in how he understood psychiatric nursing: technical tasks were necessary, but caring for long-stay patients also required attention to experiences they found difficult to express. Across participants’ accounts, learning to observe changes in behavior, anticipate risk, interpret distress, and adjust responses represented the development of clinical judgment specific to psychiatric nursing.

#### Learning with the team

Professional growth was also embedded in relationships with colleagues. Senior nurses and ward teams helped participants learn how to read patient behavior, anticipate risk, and respond collectively. Participant N16 described how experienced colleagues guided him after he transferred to an acute male ward:

Experienced nurses taught me that when a patient’s mood suddenly rises, when his voice becomes louder, or when he keeps walking around restlessly, conflict may happen. You need to prevent it early. With these patients, you should never go alone; you need staff from different roles to work together to calm and control the situation. (N16, 31–40 years, 6–10 years’ experience)

Participant N16’s account shows that team learning was practical and situated, involving specific ways of noticing, preventing, and managing escalation.

Collegial support also helped participants tolerate responsibility and remain in the work. Some recalled being supported during early independent duties, while others described ward teams as sources of practical and emotional help beyond routine clinical tasks. In this sense, the ward served as both a workplace and a community through which participants gained confidence, safety, and belonging.

#### Finding meaning in care

Participants often found professional meaning in small, everyday acts that responded to patients’ immediate needs rather than in exceptional achievements. Small gestures could become important because they were received by patients as signs of being noticed and valued. Participant N16 described buying snacks for a patient whose family had not visited: *It was only a small thing. But the patient felt very warmed by it and wrote me a thank-you letter when he was discharged. For us*,* it was just a small effort*,* but patients may remember it* (N16, 31–40 years, 6–10 years’ experience). His account shows how buying snacks for a patient who had not received a family visit became emotionally significant when the patient experienced the gesture as recognition and concern.

Meaning was also built through helping patients and families make sense of illness and treatment. Participant N09 described repeated conversations with a family who had initially resisted admission, gradually helping them move toward trust. He connected this work to a broader sense of professional purpose:

After I understood what psychiatric nursing was, I began to feel that my life goal was to help patients solve problems. Helping them and their families gives me a sense of calm, as if I have lived up to this profession. (N09, 21–30 years, 1–5 years’ experience)

Professional meaning, here, came not from clinical outcomes but from sustained relational work.

Participant N03’s account introduced a more ambivalent dimension. After buying food for a long-stay patient, he was unexpectedly attacked; the patient’s apology the next day reframed the encounter: *I felt relieved. I realized that this work is not only a job or a salary* (N03, 31–40 years, over 15 years’ experience). For N03, meaning emerged through the patient’s apology, which allowed him to reinterpret a painful encounter and recognize significance in the work beyond salary alone.

### Theme 3. Strength in caring

Theme 3 describes how participants developed a form of strength within psychiatric nursing. Physical strength was experienced as a practical resource in acute-risk situations, particularly when participants were expected to protect patients and colleagues. However, protective strength was not understood simply in terms of force, dominance, or stereotypical notions of male physical toughness. Effective strength also required restraint, calmness, sensitive communication, and relational understanding in caring for vulnerable patients.

#### Physical strength as a resource

Participants described male physical presence as a practical resource in specific clinical situations. Participant N11 described male physical presence as particularly useful in emergencies:

When restraining impulsive patients, when patients fall, become aggressive, or attempt suicide, male nurses are really needed. I need to take on greater responsibility, protect patients, and carry the part of the work where male physical advantage can be used. (N11, 21–30 years, 1–5 years’ experience)

Physical strength, in his account, was framed as a situated responsibility toward patient safety, distinct from any claim to authority or dominance.

Participants also recognized the limits of this advantage. Participant N15 noted that being male could help in situations involving self-harm, impulsive behavior, or physical control, while also identifying a limitation:

As a man, you carry a certain pressure, a slight sense of intimidation, and we are better at responding to sudden behavior, so male nurses do have an advantage there. But female colleagues are better at empathy, so in psychological care and in contact with patients, it is easier for them to reach the patient’s inner world. Men are relatively weaker in this, and I think that is a gap for us. (N15, 31–40 years, 6–10 years’ experience)

His account illustrates how gendered beliefs shaped professional self-appraisal: he associated male nurses with physical presence and rapid response, and female nurses with empathy and emotional attunement. Within this framing, relational care became an area in which he believed male nurses needed further development.

#### Protective strength

A key feature of participants’ accounts was the distinction between strength and retaliation. Participants recalled that, early in their careers, being hit or threatened by patients could evoke anger, humiliation, and even an impulse to strike back. Participant N14 spoke frankly about how this distinction was learned rather than given: *When patients hit you impulsively*,* sometimes you feel the urge to hit back. Earlier in my career*,* to be honest*,* I sometimes struggled to manage that feeling* (N14, 41–50 years, over 15 years’ experience). His account shows that professional restraint was not automatic. Across participants’ accounts, restraint developed over time through clinical experience and a growing understanding of patients’ illness and vulnerability. Participant N16 provided a more specific account of training, linking greater confidence in managing aggressive behavior to hospital-supported learning in conflict de-escalation, breakaway techniques, and violence prevention.

Physical strength alone was also seen as insufficient in crisis situations. Participant N05 described how, during night shifts, when fewer staff were routinely present on the ward, and additional support might need to be called in, professional skills and verbal de-escalation were especially important:

At night, if a patient becomes impulsive, we still need to use professional skills, not necessarily confront him directly. We need to verbally calm him and wait for more people to help (N05, 21–30 years, 11–15 years’ experience).

Strength, here, meant knowing when to hold back as much as when to act. Participant N05 also challenged assumptions about male invulnerability:

Others may think that because I am tall and big, I can handle anything. But actually, in some situations, it is still very difficult. I am not different from others.

The admission reframes what physical strength meant in practice. It was less a physical given than a capacity that required clinical judgment and emotional discipline to be of any use.

### Strength with relational care

Some accounts explicitly treated physical readiness and relational sensitivity as complementary rather than opposed. Participant N16 captured this integration clearly:

Men can also be rough in manner but attentive to detail. Nursing mainly requires carefulness and the ability to care for patients. At the same time, in other aspects, such as transferring patients from bed to chair, men may have more physical strength. (N16, 31–40 years, 6–10 years’ experience)

His account presents male psychiatric nursing as a combination of physical capacity, attentiveness, and responsibility.

Other participants similarly resisted reducing their contribution to physical labor. They described psychiatric nursing as involving psychological rehabilitation, relationship-building, and sustained trust with patients.

Participants described situations in which gender limited their direct involvement in caring for female patients, particularly when privacy, bodily exposure, or close physical contact were involved. They sometimes had to balance immediate safety concerns with patients’ comfort and dignity and seek assistance from female colleagues when appropriate. Participant N07 explained:

Sometimes a young female patient stays in the bathroom and may be at risk of self-harm, but it is difficult for me to go in directly. If it were a male patient, I would enter immediately. For an electrocardiogram with a young female patient, I usually ask a female nurse to help. (N07, 21–30 years, 1–5 years’ experience)

These experiences showed that relational care also involved recognizing when male physical presence could become a disadvantage and adapting care to protect both patient safety and privacy. Strength in caring, across these accounts, was a professional orientation developed through restraint and relational work, not a fixed male trait.

## Discussion

This study examined how male psychiatric nurses in China developed professional identity across their careers. Its central contribution is to show that professional identity developed gradually and unevenly rather than being established at entry or demonstrated simply by continued employment. This process involved navigating pragmatic entry, early strain, and double stigma; learning to understand patients and professional roles through relationships; and negotiating gendered meanings of strength and caring. Clinical confidence, commitment, and meaning in care contributed to professional identity development but were not equivalent to it. Longer-term professional identification also varied across accounts. The discussion examines these findings across the occupational journey, through relational learning, and through the negotiation of gendered recognition and the professional meaning of strength.

### Professional identity development across the occupational journey

The theme *Stumbling into commitment* showed that professional identity developed through continued engagement rather than being established at entry. Its four subthemes—*Pragmatic entry*,* Early shock and strain*,* Negotiating double stigma*, and *Commitment through accumulation*—describe how an uncertain occupational position was tested, negotiated, and often consolidated through experience.

Many participants entered psychiatric nursing without a strong initial preference for the specialty. Their decisions reflected available positions, family advice, examination results, and stable hospital employment. This accords with studies showing that employment prospects and job security influence men’s entry into nursing [7, 8]. A study of psychiatric nurses in Ghana described a broadly positive trajectory from little initial interest in the specialty to later appreciation following education and clinical exposure [23]. Among the male psychiatric nurses in the present study, the trajectory was more varied. Some participants developed strong commitment through accumulated experience, whereas others remained primarily oriented toward job stability or described occupational fatigue. These findings suggest that pragmatic entry can be followed by professional identification, but not uniformly.

Early ward demands and clinical risk could be unsettling. Participants also encountered gender stereotypes positioning nursing as women’s work [7] and stigma attached to mental illness and psychiatric care [3, 23]. Some avoided identifying themselves directly as psychiatric nurses or described their occupation more broadly. These pressures shaped how participants positioned themselves socially in relation to their work and constituted a double stigma, although the data do not show whether the two forms intensified each other.

Commitment developed through accumulated experience rather than a single turning point. In one Chinese study, commitment to nursing was described as a dynamic and sometimes nonlinear process from difficult transition and thoughts of leaving to adaptation and role acceptance, while most participants still viewed nursing primarily as a means of earning a living [24]. This suggests that settling into the role can coexist with pragmatic attachment to the occupation. Research comparing male nurses in mainland China and Macau also found different long-term orientations. Mainland participants often remained uncertain about their future and sometimes pursued promotion as a route away from frontline care [14]. The uneven pattern observed in the present study is also consistent with evidence that junior male nurses reported lower professional identity than male nursing students, with heavy workload and gender stereotypes associated with lower identity [16].

Remaining in nursing may therefore reflect adaptation, economic considerations, career plans, or professional commitment. The present study shows how these different orientations appeared in psychiatric nursing. Stable public-hospital employment gave participants time to develop competence, receive recognition, and find meaning in their work, but it did not lead everyone to identify with the profession in the same way. Retention and professional identification were related but distinct in participants’ accounts.

### Relational contexts shaping professional identity

The theme *Growing through relationships* showed that professional identity developed as participants learned what psychiatric nursing required and recognized the value of their contribution. Its three subthemes—*Developing relational understanding*,* Learning with the team*, and *Finding meaning in care*—show how relationships with patients and colleagues shaped participants’ understanding of patients, practice, and themselves as psychiatric nurses.

Contact with patients helped participants interpret behavior and distress rather than respond only to observable actions. Working with experienced nurses helped them assess risk and calibrate their responses. These findings accord with research showing that mental health nursing identity develops through relationships with patients, role models, and the acquisition of specialist knowledge [25, 26]. They are also consistent with accounts from experienced male mental health nurses that emphasize compassion, teamwork, and professional fulfillment [27]. The present findings clarify how this development occurred. Relational understanding and context-sensitive judgment accumulated through everyday clinical encounters.

Aggression and violence were particularly important in this learning. Their prominence should be understood in the context of the high prevalence of workplace violence in Chinese psychiatric settings, estimated at approximately 78% in a meta-analysis [28]. In a national survey, 81% of psychiatric professionals reported exposure in the previous year [29]. Participants gradually interpreted aggression, resistance, withdrawal, and silence in relation to patients’ symptoms, distress, and the ward environment. One participant’s account suggests that structured training may reinforce the preventive judgment developed through clinical experience and team-based learning. Prior research indicates that psychiatric nurses may regard violence as routine while continuing to experience fear and lasting effects [30]. The emotional toll remained evident here, suggesting that participants developed contextual judgment rather than simply becoming inured to violence. Because the study included only male nurses, it remains unclear whether this process was specific to men or reflected psychiatric nursing more broadly.

Team relationships and patients’ responses extended this learning. Senior nurses helped participants recognize risk and judge when support was needed, consistent with evidence that supportive working relationships and skill development help sustain mental health nurses in practice [31]. Patients’ gratitude, trust, and responses to consistent attention helped participants recognize the value of ordinary care, even when recovery was slow. This accords with research linking meaningful nursing work to recognition, trusting relationships, and awareness of one’s contribution [32]. These experiences connected clinical competence with belonging and professional value, contributing to how participants understood themselves as psychiatric nurses.

### Gendered recognition and the professional meaning of strength

The theme *Strength in caring* showed that gendered recognition shaped which aspects of participants’ work were visible and professionally valued. Its three subthemes—*Physical strength as a resource*,* Protective strength*, and *Strength with relational care*—showed that physical capability gained professional meaning when combined with restraint, clinical judgment, teamwork, and relational care.

Participants were particularly visible when managing aggression, preventing harm, and maintaining ward safety. This accords with research showing that male nurses may receive recognition for perceived physical or technical strengths while continuing to encounter gender stereotypes [7, 8, 33]. Research on men in mental health nursing has cautioned against defining their contribution primarily through physical strength and crisis management [13]. The present findings reveal a tension within this recognition: perceived competence in high-risk situations strengthened participants’ sense of usefulness and acceptance but could narrow the range of contributions for which they were recognized.

Physical strength was described as a situational resource rather than a general measure of professional value. Its usefulness depended on patients’ needs, the level of risk, and its combination with communication, judgment, and coordinated action. In protective situations, strength acquired professional value through restraint and timing rather than force alone. This finding complicates gendered expectations in mental health nursing that associate men with physical tasks and women with relational care [25], as well as broader masculinity stereotypes that position men as physically capable and suited to crisis-oriented work [7, 8, 13]. One participant reproduced these stereotypes by linking male nurses to physical presence and rapid response and female nurses to empathy and emotional attunement. His account illustrates how gendered beliefs informed the appraisal of male nurses’ professional strengths and limitations. Such beliefs may provide recognition for physical contributions while obscuring relational competencies. Participants did not directly describe physical strength as an obstacle; this narrowing effect is an interpretation of how professional recognition was organized in their accounts.

Participants’ experiences also complicated a simple contrast between physical and relational care. Effective responses to high-risk situations required communication, restraint, teamwork, and trust. When caring for female patients in situations involving privacy, bodily exposure, or close physical contact, participants sometimes judged direct involvement to be inappropriate and sought assistance from female colleagues, a limitation also reported among male nurses in China [34]. Recognizing when physical presence was useful and when it could become a disadvantage was itself part of context-sensitive caring judgment. These findings challenge role definitions centered on physical control by locating professional value in the judgment governing when and how strength was used.

Gender was expressed differently across the three themes. In *Stumbling into commitment*, it appeared through the double stigma of being men in nursing and working in psychiatry. In *Growing through relationships*, participants described learning from patients and colleagues largely in professional and relational terms, without explicitly framing these experiences through gender. In *Strength in caring*, participants directly articulated gendered expectations through physical capability, safety work, intimate care, and the allocation of responsibilities. Across these accounts, gender entered professional identity development through particular situations, including social disclosure, physical presence, and role allocation.

### Strengths and limitations

A key strength was the methodological coherence between the exploratory aim, semi-structured interviews, and reflexive thematic analysis, which enabled interpretation of shared patterns while preserving variation across participants’ accounts [18, 20]. Participants varied in age and length of psychiatric nursing experience, providing perspectives from different career stages. Detailed reporting of the study context and participants’ accounts allows readers to assess the transferability of the findings to other settings [17, 22]. Conducting the study in a specialist psychiatric hospital also enabled close examination of patient relationships, workplace violence, team learning, and the situational use of physical strength in psychiatric care.

Several limitations should be considered. Convenience sampling from one specialist psychiatric hospital may have preferentially included nurses willing to discuss their experiences, and the findings remain context-specific. The sample excluded men who had transferred or left psychiatric nursing, limiting insight into more difficult career pathways. The cross-sectional design captured accounts at one time and could not demonstrate change across participants’ careers. Finally, the male-only sample and absence of a formal masculinity framework limit conclusions about which experiences were gender-specific and how masculine norms were reproduced, negotiated, or resisted. Such a framework might have influenced the research questions, sampling, interviews, and analysis. The gender-related findings should therefore be understood as a sociocultural dimension of professional identity development rather than a systematic account of masculinity. Future comparative and theory-informed research could address these limitations.

## Implications

These findings have implications for supporting male psychiatric nurses across career stages. During entry and early adaptation, structured orientation should address ward risk, occupational stigma, and the emotional impact of aggression and violence, with timely support after distressing incidents. Mentorship, clinical supervision, and team-based learning may strengthen relational understanding and context-sensitive judgment [35, 36]. Structured education can link violence risk assessment with aggression prevention, while de-escalation training may improve staff knowledge, confidence, and observed performance in managing aggression [37, 38]. Managers should recognize male nurses’ contributions to ward safety without routinely positioning them as default crisis responders, which may narrow their professional roles and obscure their relational and caring competencies. Because continued employment reflected different degrees of professional identification, longer-term support should also attend to pragmatic retention, declining motivation, and occupational fatigue. Multi-site and longitudinal research could examine how professional identity develops across institutional settings and career stages.

## Conclusion

This study shows that professional identity among male psychiatric nurses developed gradually and unevenly through occupational experience, relationships, and the social context of psychiatric nursing. Pragmatic entry could develop into commitment as participants adapted to ward demands, gained confidence, and accumulated meaningful experiences, although continued employment reflected different levels of professional identification. Relationships with patients and colleagues fostered relational understanding, clinical judgment, belonging, and recognition of their contribution. Being regarded as physically capable and able to manage challenging situations contributed to some participants’ sense of professional value, particularly when these capacities were combined with clinical judgment, communication, teamwork, and relational care. At the same time, gendered expectations risked reducing male psychiatric nurses to physical and safety-related roles and obscuring their broader caring and clinical competencies. These findings characterize professional identity development as an evolving occupational, relational, and sociocultural process. Support for male psychiatric nurses should address early adaptation, relational learning, gendered role expectations, and differing longer-term levels of commitment and occupational fatigue.

## Supplementary Information

Below is the link to the electronic supplementary material.

Supplementary Material 1

## Data Availability

The datasets generated and analyzed during the current study are not publicly available due to the qualitative nature of the data and the need to protect participant confidentiality and privacy. Relevant de-identified excerpts are included in the manuscript. Raw de-identified data may be available from the corresponding authors upon reasonable request and subject to ethical approval and institutional permission.

## Declarations

### Study registration

Chinese Clinical Trial Registry (ChiCTR2400091574), Registered 30 October 2024.

### AI usage

The authors used ChatGPT to assist with the initial translation of selected quotations and with language editing and refinement. The authors reviewed and revised all AI-assisted outputs and take full responsibility for the final content.
