# Supplementary material for: Professional identity development among male psychiatric nurses in China: a qualitative study
Source: BMC Nurs. 2026 Sep 21;25:874. doi: 10.1186/s12912-026-05364-7 (PMC13595714; doi:10.1186/s12912-026-05364-7)
Supplement: Supplementary file 1 [file 12912_2026_5364_MOESM1_ESM.docx]

**Supplementary file 1**

**Semi-structured interview guide**

This semi-structured interview guide was developed specifically for this study. The interviews were conducted flexibly, with follow-up prompts and probing questions used to clarify participants’ responses and encourage them to elaborate on relevant experiences.

**Interview questions**

1. Could you describe your experience of becoming a psychiatric nurse?
2. How do you view your profession? Has your view changed over time? How do your family members or friends view your work?
3. What challenges or difficulties have you encountered at different stages of your career? How did you respond to these challenges? What kinds of support did you receive? How did these experiences influence your career development?
4. How do you understand the relationship between your career development and personal growth?
5. Could you talk about your future career plans? What kinds of support would help you better achieve these plans?
6. Is there anything else that is important to your career development but has not been discussed?

**Examples of follow-up prompts and probing questions**

Can you tell me more about it?

Could you give me some examples?

How did you feel about that experience?

What did that experience mean to you?

Did this change over time? If so, how?

What do you mean by [the participant’s own word or expression]?
